# Associative patterns among anaerobic fungi, methanogenic archaea, and bacterial communities in response to changes in diet and age in the rumen of dairy cows

**DOI:** 10.3389/fmicb.2015.00781

**Published:** 2015-07-31

**Authors:** Sanjay Kumar, Nagaraju Indugu, Bonnie Vecchiarelli, Dipti W. Pitta

**Affiliations:** Agriculture Systems and Microbial Genomics Laboratory, Department of Clinical Studies, New Bolton Center, School of Veterinary Medicine, University of Pennsylvania, Kennett Square, PAUSA

**Keywords:** anaerobic fungi, co-occurrence, diet, methanogenic archaea, microbiome, rumen

## Abstract

The rumen microbiome represents a complex microbial genetic web where bacteria, anaerobic rumen fungi (ARF), protozoa and archaea work in harmony contributing to the health and productivity of ruminants. We hypothesized that the rumen microbiome shifts as the dairy cow advances in lactations and these microbial changes may contribute to differences in productivity between primiparous (first lactation) and multiparous (≥second lactation) cows. To this end, we investigated shifts in the ruminal ARF and methanogenic communities in both primiparous (*n* = 5) and multiparous (*n* = 5) cows as they transitioned from a high forage to a high grain diet upon initiation of lactation. A total of 20 rumen samples were extracted for genomic DNA, amplified using archaeal and fungal specific primers, sequenced on a 454 platform and analyzed using QIIME. Community comparisons (Bray–Curtis index) revealed the effect of diet (*P* < 0.01) on ARF composition, while archaeal communities differed between primiparous and multiparous cows (*P* < 0.05). Among ARF, several lineages were unclassified, however, phylum Neocallimastigomycota showed the presence of three known genera. Abundance of *Cyllamyces* and *Caecomyces* shifted with diet, whereas *Orpinomyces* was influenced by both diet and age. *Methanobrevibacter* constituted the most dominant archaeal genus across all samples. Co-occurrence analysis incorporating taxa from bacteria, ARF and archaea revealed syntrophic interactions both within and between microbial domains in response to change in diet as well as age of dairy cows. Notably, these interactions were numerous and complex in multiparous cows, supporting our hypothesis that the rumen microbiome also matures with age to sustain the growing metabolic needs of the host. This study provides a broader picture of the ARF and methanogenic populations in the rumen of dairy cows and their co-occurrence implicates specific relationships between different microbial domains in response to diet and age.

## Introduction

Ruminants cannot degrade recalcitrant plant lignocellulosic material on their own; instead they depend on microbial communities (i.e., bacteria, anaerobic fungi, protozoa, and methanogenic archaea) that reside within their rumen (Hobson and Stewart, 1997). Although diverse, these microbial communities work together synergistically and contribute substantially to the functional attributes and health of the host. A majority of rumen microbiome studies have been directed at investigating bacterial diversity and the various factors that influence bacterial dynamics using advanced molecular tools including next generation sequencing (NGS) technology. However, our understanding of the diversity associated with anaerobic rumen fungi (ARF) and methanogens in the rumen and their functional importance is still in its infancy. Anaerobic fungi, despite their low numbers (10^6^/mL of rumen liquid), play a significant role in the degradation of ingested plant cellulosic fibers by both invasive rhizoidal growth and by producing a vast array of polysaccharide-degrading enzymes (Dagar et al., 2011; Gruninger et al., 2014; Puniya et al., 2015). A majority of these ARF belong to the order Neocallimastigales within the phylum Neocallimastigomycota (Liggenstoffer et al., 2010). Six genera (*Neocallimastix, Piromyces, Caecomyces, Orpinomyces, Anaeromyces*, and *Cyllamyces*) have been identified based on their phylogeny (Dagar et al., 2011). Recent studies (Liggenstoffer et al., 2010; Kittelmann et al., 2012) have reported the existence of novel ARF clades which cannot be classified to a taxonomic rank due to the lack of cultured representatives, indicating the need for further expansion of ARF culture collection (Griffith et al., 2009; Liggenstoffer et al., 2010; Kittelmann et al., 2012). Further, novel markers in addition to rRNA gene or ITS regions are required to better characterize ARF communities in the rumen (Griffith et al., 2009; Liggenstoffer et al., 2010; Kittelmann et al., 2012).

Ruminal archaea account for 0.3–3.3% of ruminal microbial small subunit RNA (Janssen and Kirs, 2008) and are solely responsible for methane production in the rumen. Methane emitted from ruminants accounts for a loss of 2–12% of metabolizable energy intake, and also causes an environmental problem due its large global warming potential (Johnson and Johnson, 1995; Kumar et al., 2009). Therefore, in order to develop methane mitigation strategies, an understanding of methanogenic diversity in the rumen is essential. Until recently, rumen methanogens were assigned to a few genera in the orders *Methanobacteriales, Methanomicrobiales*, and *Methanosarcinales*, within the phylum *Euryarchaeota*. However, based on 16S rRNA gene sequences, a novel group distantly related to the *Thermoplasmatales* (named rumen Cluster C; previously described as rice cluster C *Thermoplasmata*) was found to be highly abundant in ruminants (Janssen and Kirs, 2008; Poulsen et al., 2013). *Methanobrevibacter* is the most commonly encountered genus within *Methanobacteriales*. Also abundant in some ruminants is the genus *Methanomicrobium* (Chaudhary and Sirohi, 2009; Kumar et al., 2012). While other members of *Methanomicrobium* have shown abundance with culture-independent methods, they are rarely detected/ isolated with conventional approaches. The order *Methanosarcinales* comprises a group of physiologically distinct aceticlastic methanogens (Janssen, 2010), but their abundance in the rumen is low.

The rumen microbes, albeit different in phylogeny, are intrinsically linked and their symbiotic relationship is central to rumen function, particularly fiber digestion (Creevey et al., 2014). Bacteria, ARF and protozoa colonize and decompose the indigestible lignocellulosic material and in the process release hydrogen (Akin et al., 1988) which ruminal archaea utilize, as they are hydrogen scavengers (Janssen and Kirs, 2008). However, the initial colonization of fiber by ARF can facilitate a rapid fibrolytic activity by bacteria and other microbial domains (Sehgal et al., 2008), thus fiber degradation by ARF has the capability to determine the composition of other microbial communities (Kittelmann et al., 2012). Such cross-domain inter-dependency has been demonstrated in co-culture studies (Joblin et al., 1989, 2002), where Joblin et al. (1989, 2002) demonstrated synergism between cellulolytic bacteria, ARF and *Methanobrevibacter smithii* in the degradation of barley straw as well as perennial ryegrass. Recent findings from Piao et al. (2014) showed that fiber degradation was temporarily halted until hydrogen was utilized by methanogens. Therefore, in this complex genetic web of the rumen microbiome, it becomes obvious that exploring the interactions between different microbial domains is the key to manipulating rumen function and enhancing animal health and productivity. Documentation on the microbe–microbe interactions within the rumen microbial consortium is sparse and limited to the recent reports of Kittelmann et al. (2013) and Belanche et al. (2014). These authors demonstrate associations and interactions between the most dominant microbial species in the rumen and emphasize the need for further studies to validate the identified associations.

Recently, investigating the functional role of the rumen microbiome in dairy cows has become the holy grail of several scientists (Jami et al., 2013, 2014; Mao et al., 2014; Pitta et al., 2014). Hristov et al. (2012) investigated the changes in rumen bacterial, archaeal, and fungal diversity in cows supplemented with medium-chain fatty acids such as stearic, lauric and myrstic acids. The authors report that lauric acid markedly reduced protozoan numbers which indirectly influenced both bacterial and archaeal communities. Since diet is the main driver for inducing microbial shifts in the rumen, various studies have attempted to relate dietary shifts with microbial dynamics as well as with methane emissions in the rumen (Zhou and Hernandez-Sanabria, 2009, 2010; Kumar et al., 2013). In our recently published study, we demonstrated that transitioning dairy cows switching from a low energy (80% forage and 20% concentrate) to a high energy (50% forage and 50% concentrate) diet, exhibited increased *Bacteroidetes* and reduced *Firmicutes* populations (Pitta et al., 2014). Furthermore, due to significant differences in the milk yields of primiparous (first lactation; age ~2 years) and multiparous (≥second lactation; age ≥3 years) cows (Dado and Allen, 1994; Miller et al., 2006; Wathes et al., 2007; Lang et al., 2012), we hypothesized that the microbial community composition would be significantly different between the two groups of cows. As expected, our report showed that the bacterial community composition was different between primiparous and multiparous cows, indicating that the microbiome continues to evolve and mature with age (first lactation vs subsequent lactation cycles). In this study, employing sequence-based pyrosequencing technology, we investigated if the community composition of ARF and archaea is altered in response to a changing diet and differences in age of dairy cows. Further, we also investigated the associative patterns among the most abundant lineages of bacteria, ARF and archaea, using co-occurrence analysis.

## Materials and Methods

### Animals

The present study included ten dairy cows of Holstein breed, divided into two study groups (primiparous: *n* = 5 and multiparous: *n* = 5), selected randomly from the dairy herd. The dairy cows were housed at Marshak Dairy, New Bolton Center according to ethics committee input and IACUC standards for the University of Pennsylvania.

### Dietary Composition

The dairy cows were maintained on a dry cow ration composed of 80% forages and 20% concentrate (D1) during the pre-calving period. Dairy cows received an energy rich diet composed of 50% forage and 50% concentrate (D2) after calving. The greater forage inclusion in the dry cow diet compared to the high-energy diet was reflected in higher NDF (43.7% versus 33.1%, respectively), lignin (3.2% versus 2.6%, respectively) lower starch (21.9% versus 27.2%, respectively) and CP (14.7% versus 17.2%, respectively) contents (**Table 1**).

**Table 1:** Nutrient composition of high forage (Pre-calving: D1) and high energy (Post-calving: D2) diets.

	D1	D2
**Ingredient composition (% DM)**		
Corn silage	43.2	36.7
Rye silage	17.3	11.3
Sorghum	–	
Grass-alfalfa silage	–	
Grass hay	19.2	
Fine ground corn	9.9	21.5
Soybean meal	4.1	8.5
AminoPlus	2.0	5.0
Blood meal	3.1	0.4
Dry corn distillers	–	2.2
Soybean hulls	–	6.0
Wheat middlings	–	3.4
Vitamin-mineral mix	0.37	0.16
**Chemical composition (% DM basis)**		
Forage	78.7	48.0
CP	14.6	16.8
Soluble protein, % CP	34.0	31.3
RDP	9.3	10.3
ADF	28.2	19.6
NDF	44.0	32.5
Sugar	2.6	3.5
Starch	21.9	29.1
Fat	3.1	3.9

### Rumen Sampling

The rumen contents were collected approximately 2–3 h after the morning feeding by the stomach tube method (Lodge-Ivey et al., 2009). Each cow was sampled twice, i.e., 4 weeks before calving (D1) and 1–5 days after calving (D2). The initial 200 mL of rumen contents was discarded to avoid any contamination with saliva and subsequent samples (~250 mL) were collected, divided into aliquots in 15 mL polypropylene tubes and snap frozen in liquid nitrogen. The samples were then transported to the laboratory and kept frozen at -80°C until processing for DNA extraction.

### DNA Extraction, PCR, and Pyrosequencing

The genomic DNA was extracted from the rumen samples using the PSP Spin Stool DNA Plus Kit (Invitek, Berlin, Germany) as per the method of Mckenna et al. (2008). The extracted DNA was subjected to PCR amplification in quadruplicate using the Accuprime Taq DNA polymerase System (Invitrogen, Carlsbad, CA, USA). Archaeal (16S rDNA; 958aF-deg: 5′-AATTGGAKTCAACGCCKGR-3′ and 1378aR: 5′-TGTGTGCAAGGAGCAGGGAC-3′) and fungal (internal transcribed spacer, ITS1-F: 5′-CTTGGTCATTTAGAGGAAGTAA-3′ and ITS1-R: 5′-GCTGCGTTCTTCATCGATGC-3′) specific primers and thermal cycling conditions were similar to those described by Hoffmann et al. (2013). The resulting amplified PCR products were bead purified using a Beckman Coulter Agencourt AMPure XP Beads (Beckman-Coulter, Pasadena, CA, USA) as described by Hoffmann et al. (2013). The amplicons generated for each sample were pooled in equimolar concentrations and subjected to pyrosequencing using a 454 Junior Roche Platform (GS FLX Titanium).

### Data Analysis

The 16S pyrosequence reads for archaea were analyzed using the QIIME pipeline (Caporaso et al., 2010b), followed by statistical analysis in R Core Team (2013). Reads were discarded if they did not match the expected sample-specific barcode and 16S primer sequences were shorter than 200 bp or longer than 800 bp, or contained a homopolymer sequence in excess of 6 bp. Operational taxonomic units (OTUs) were formed at 97% similarity using UCLUST (Edgar, 2010).

Representative sequences from each OTU were aligned to 16S reference sequences with PyNAST (Caporaso et al., 2010a) and used to infer a phylogenetic tree with FastTree (Price et al., 2010). Taxonomic assignments within the GreenGenes taxonomy (12/10 release; McDonald et al., 2011) were generated using the RDP Classifier version 2.2 (Wang et al., 2007). For ARF, reads were quality trimmed as mentioned above. OTUs were picked using open-reference OTU picking method and the its_12_11_otus reference taxonomy, provided by the UNITE database (https://unite.ut.ee). Species richness (chao1), diversity (Shannon Diversity index) and observed OTUs were chosen as indices for alpha-diversity (within sample comparison) and calculated for each of the archaeal and ARF communities as per the scripts built in QIIME (Caporaso et al., 2010a). A non-parametric two sample *t*-test was applied to compare the alpha diversities using the default number of Monte Carlo permutations (999). A non-parametric permutational multivariate ANOVA test (Anderson, 2001), implemented in the vegan package of R (Oksanen et al., 2013), was used to test the effects of diet and study group on overall community composition, as measured by the Bray–Curtis distance matrix. The Dice index (Dice, 1945) was used to determine the co-occurrence of genera across bacteria, fungi and archaea. Genera were considered present in a sample if its sequence proportion was at least 0.01 for bacteria and archaea and 0.001 for fungi.

## Results

### Fungal and Methanogenic Archaea Community Profiles

Nearly 35,582 reads were sequenced for rumen fungi, which yielded 310 representative OTUs annotated to five phyla (Supplementary Table S1) such as Ascomycota (27%), Basidiomycota (3%), Neocallimastigomycota (1%), Zygomycota (<1%), and unclassified (68%). Although the percent of sequences that were categorized in Neocallimastigomycota was low, we were interested in determining the diversity of lineages in this strictly anaerobic phylum in the rumen microbiome. In this phylum, we identified three known genera, namely *Cyllamyces, Caecomyces*, and *Orpinomyces*. For Archaea, approximately 18,317 reads were annotated to 180 OTUs with a majority of these OTUs annotated to the phylum Euryarchaeota. In this phylum, genus *Methanobrevibacter* was the most dominant (96%) followed by genus *Methanosphaera*, which contributed 3% of archaeal abundance. Lineages from Vadin CA11 (order *Thermoplasmatales*) was also identified but contributed to <1% of abundance.

### Species Richness and Diversity

**FIGURE 1:**
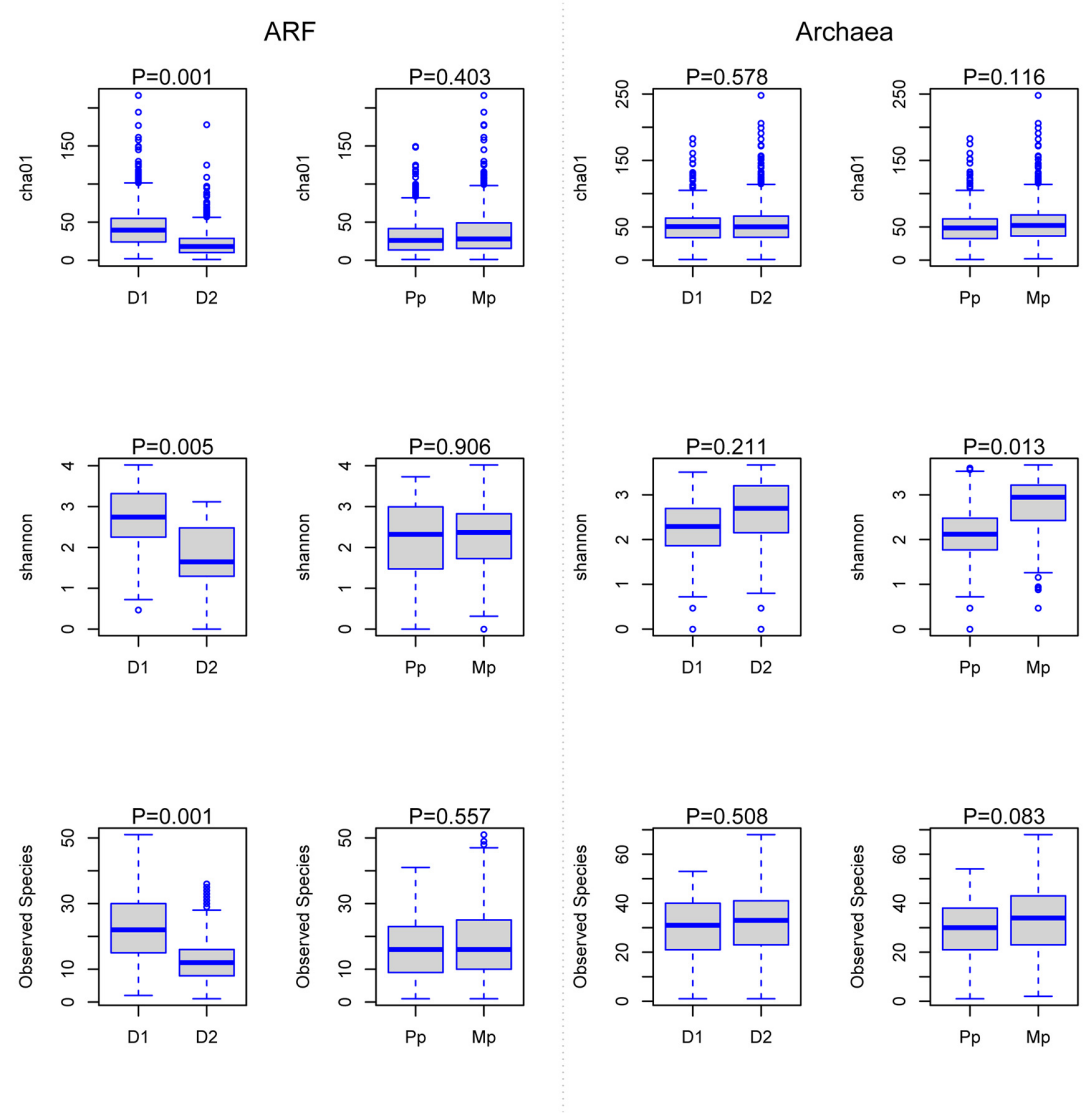
**Changes in anaerobic rumen fungi (ARF) and methanogenic archaeal richness, species diversity, and OTUs.** Boxes represent the interquartile range (IQR) between the first and third quartiles (25th and 75th percentiles, respectively), and the horizontal line inside the box defines the median. Whiskers represent the lowest and highest values within 1.5 times the IQR from the first and third quartiles, respectively. D1, diet with 80% forage; D2, diet with 50% forage; Pp, primiparous cows and Mp, multiparous cows; ARF, anaerobic rumen fungi.

The number of species level taxa, measured by chao1 and observed species (richness estimates) and distribution of these taxa across all samples (Shannon diversity index) for ARF communities, were much higher (*P* < 0.05) on D1 diets compared to D2 diets, while no differences were evident between primiparous and multiparous cows. In contrast, archaeal diversity was not influenced by dietary composition; however, Shannon diversity index was higher (*P* < 0.05) in multiparous cows than primiparous cows (**Figure 1**).

### Influence of Age and Dietary Factors on ARF and Archaeal Community Composition

**FIGURE 2:**
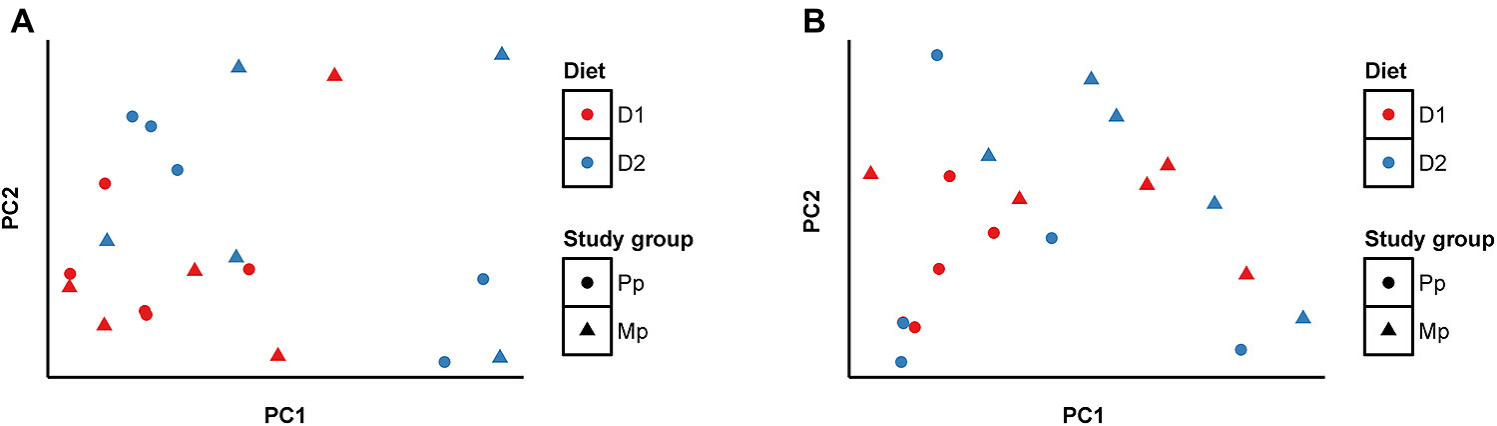
**Principal coordinate analysis (PCoA) based on Bray-Curtis distances **(A)** ARF, **(B)** Methanogenic Archaea.** Sample identifiers are same as used in **Figure 1**.

Bray–Curtis distances were calculated for each pair of ARF and archaea communities and were analyzed using principal coordinate analysis (PCoA), and are presented as a function of diets and age (**Figures 2A,B**). Comparing the D1 and D2 diets, the composition of ARF communities was significantly different (*P* < 0.01); however, no such differences were evident between primiparous and multiparous cows. In contrast, the archaeal community was resistant to changes in diet but substantial differences (*p* < 0.05) were noticed between primiparous and multiparous cows.

### Phylogenetic Diversity

**FIGURE 3:**
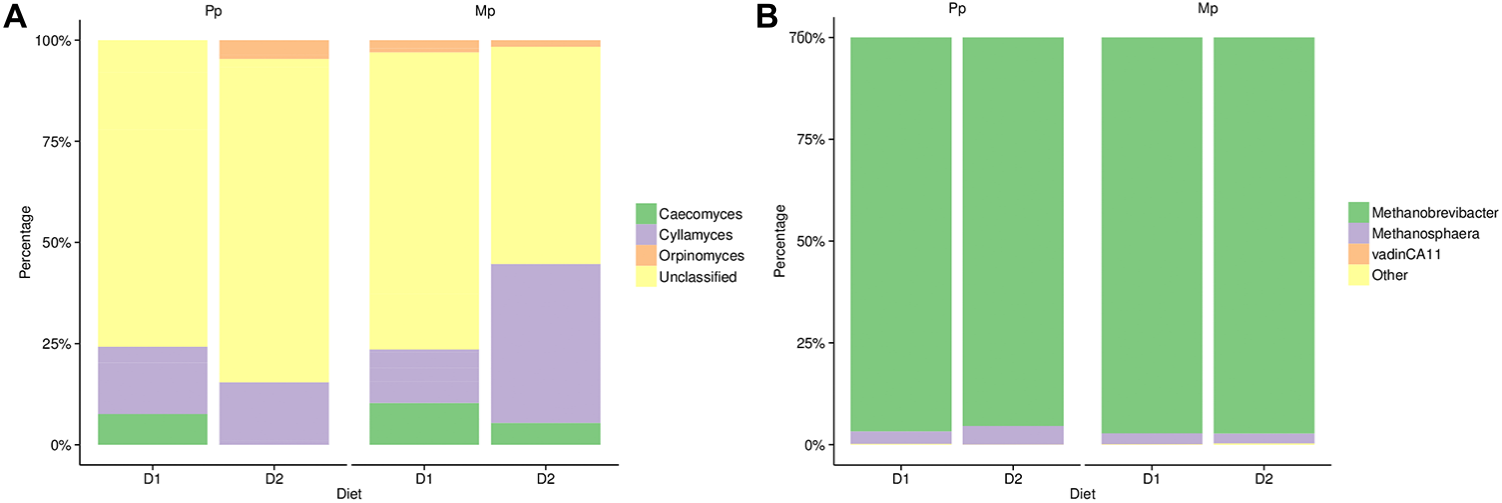
**Phylogenetic composition of **(A)** anaerobic fungi and **(B)** Methanogenic Archaea based on study group and diet.** Sample identifiers are same as used in **Figure 1**.

In the family *Neocallimastigaceae* we identified three known major genera (*Caeocomyces, Cyllamyces*, and *Orpinomyces*) in addition to the large contribution (40–75%) of unclassified members (**Figure 3A**). In primiparous cows, *Caecomyces* and *Cyllamyces* together constituted 25% of the total identified anaerobic fungi in cows fed the D1 diet. In the D2 diet the proportion of *Cyllamyces* remained unchanged; however, *Caeocomyces* was completely replaced by *Orpinomyces* and other unclassified genera. Multiparous cows were similar to primiparous cows on D1 diets except for the presence of *Orpinomyces* (~5%). However, on the D2 diet, the proportion of the genus *Cyllamyces* increased substantially (45%) at the expense of *Caecomyces, Orpinomyces*, and unclassified *Neocallimastigaceae* taxa. Among archaea, the phylum *Euryarchaeota* included Vadin CA11 (0.01–0.07%), along with the genera *Methanobrevibacter* and *Methanosphaera* which together constituted >98% of the archaeal abundance. In both diets (D1 and D2) and study groups (primiparous and multiparous), *Methanobrevibacter* was the most dominant genus accounting for nearly 94–98% abundance. Although genus *Methanosphaera* was present in both groups, its abundance was slightly higher (4.5%) in primiparous than in multiparous cows (3.0%), in particular those on the D2 diet (**Figure 3B**).

### Co-Occurrence between Different Microbial Domains in the Rumen Microbiome

**FIGURE 4:**
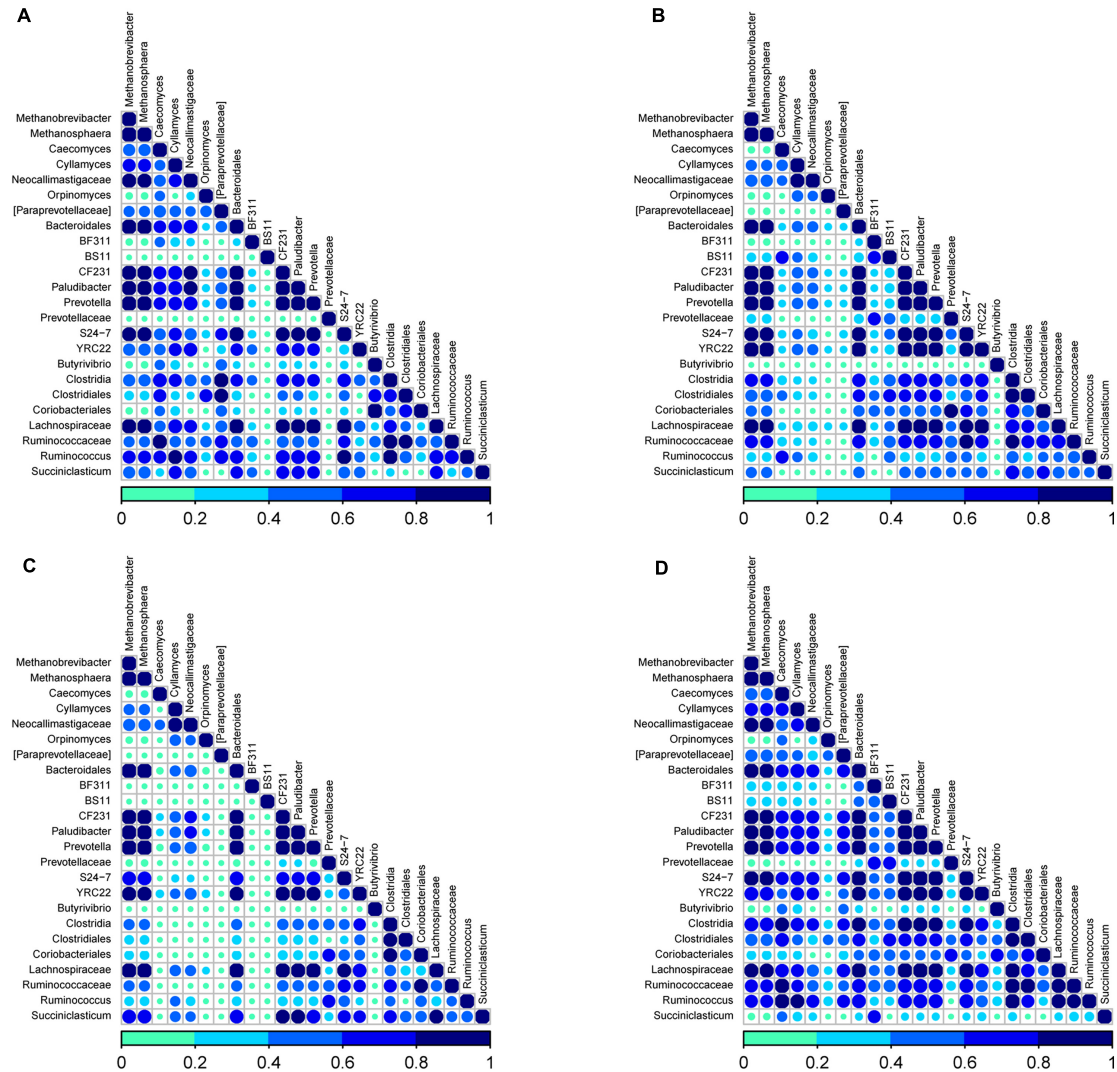
**Analysis of co-occurrence among microbial lineages scored using the Dice index **(A)** diet D1, **(B)** diet D2, **(C)** primiparous cows, and **(D)** multiparous cows**. Dice indexes across all genera for bacteria and archaea pairs present at a proportion = 0.01 and for fungi at a proportion = 0.001 are shown as a heatmap. Co-occurrence is shown by the color code (navy blue: high co-occurrence, sky blue: moderate co-occurrence; green: low co-occurrence) at the bottom. Sample identifiers are same as used in **Figure 1**.

As rumen microbes work synergistically to perform various metabolic activities in the rumen, we sought to determine the associative interactions between bacteria, ARF and archaea using co-occurrence analysis based on Dice index. For this co-occurrence analysis, we used previously published data on the most abundant genera (>1%) from rumen bacterial communities in the same 10 cows (Pitta et al., 2014) along with the ARF and archaea sequencing data from the present study. We analyzed a total of 28 genera; 22 from bacteria, 4 from ARF and 2 genera from archaea. Associations were presented individually for D1 and D2 diets (**Figures 4A,B**) and also for primiparous and multiparous cows (**Figures 4C,D**). Co-occurrence is shown by the color code (navy blue: high co-occurrence, sky blue: moderate co-occurrence; green: low co-occurrence).

In the rumen of cows on the D1diet, comprised of 75% forage, most of the genera (75%) were found to potentiate each other irrespective of the microbial domain. Notably, an unclassified genus of *Prevotellaceae* (Bacteroidetes), *Butyrivibrio*, BS11, BF311 (Firmicutes) and *Orpinomyces* (ARF) appeared to have little or no interaction with any other selected genera. In the bacterial domain, members of Bacteroidetes (*Prevotella, Bacteroidales* and *Paludibacter*) and Firmicutes (*Succiniclasticum, Lachnospiraceae, Ruminococcus*, and *Clostridia*) showed high co-occurrence with each other and with methanogenic genera and ARF (*Caecomyces, Cyllamyces*). Among methanogens, *Methanobreivibacter* and *Methanosphaera* co-occurred with each other as well as with the abovementioned ARF and bacterial genera. Within ARF, *Caecomyces, Cyllamyces*, and unclassified lineages of *Neocallimastigaceae* co-occurred with each other and the abovementioned members of the bacteria and archaea domains.

With the change to the D2 diet (reduced forage proportion and greater concentrate; 50% each) the syntrophic interactions between microbial taxa that appeared very strong on D1 diets have diminished (**Figure 4B**). The most distinct loss of interactions was observed within ARF and between ARF and other domains. Except for the interactions between a few genera from Bacteroidetes, *Lachnospiraceae* from Firmicutes and both genera from archaea, all other patterns described from D1 diets seemed to fade out when cows were switched to D2 diets. These results reveal that dietary components can have a huge impact on the associative patterns between different domains in the rumen microbiome.

Interesting associations were observed in the co-occurrence analysis performed for primiparous and multiparous cows. In primiparous cows (**Figure 4C**), only five genera from bacteria and two genera from archaea showed high to moderate (1 to 0.5 on the scale) co-occurrence, while genera from Firmicutes and ARF showed low or no co-occurrence with other genera. However, in multiparous cows there seemed to be a myriad of associative patterns indicating syntrophic interactions across all microbial domains (**Figure 4D**). These results reveal that the extent of co-occurrence among microbial domains in young ruminants is naive but as dairy cows advance in lactations with improvements in metabolic efficiency, there is a greater interdependency across microbial domains in the rumen microbiome.

## Discussion

The purpose of this study was to investigate the ecology of ARF and methanogens in dairy cows and determine if age and changes in dietary composition could significantly influence these microbial domains. We found that dietary components, particularly the amount of structural carbohydrates, facilitated diverse ARF populations, while methanogens were resistant to these dietary changes. Co-occurrence analysis revealed associative patterns among bacteria, ARF and fungi which appear to be strong on a high forage diet. This study also revealed that co-occurrence both within and between microbial domains was non-existent or limited to only a few microbial taxa in primiparous cows; however, these expanded to multiple microbial species in multiparous cows, thus supporting our hypothesis that the rumen microbiome continues to evolve as dairy cows advance through lactations.

### Diversity of Anaerobic Rumen Fungi

In our study, we employed ITS-1 based primers for ARF characterization using a pyrosequencing approach. Although ITS is polymorphic and homoplasious in nature (Dagar et al., 2011; Kittelmann et al., 2012), it is still an accepted biomarker for elucidating community composition of ARF (Tuckwell et al., 2005; Edwards et al., 2008; Sundset et al., 2009; Liggenstoffer et al., 2010; Nicholson et al., 2010). Recent metagenomic approach-based studies have used the ITS-1 region to describe ARF diversity in the gastrointestinal tract of ruminants (Liggenstoffer et al., 2010; Kittelmann et al., 2012, 2013), who reported a greater diversity and the presence of novel clades of ARF.

Another finding in our study is the large percentage (68%) of unclassified sequences which may potentially contain several novel fungal lineages similar to the studies by Liggenstoffer et al. (2010) and Nicholson et al. (2010). The plausible argument for a high percent of “unclassified” or “other” sequences is due to the lack of a well-structured database to assign taxonomy rank to the sequenced reads (Liggenstoffer et al., 2010; Fernando, 2012; Fouts et al., 2012). Although previous research established a rumen fungal phylogeny based on the six known genera, several sequences deposited in the *GenBank* database were mis-annotated (Fliegerova et al., 2010). The report of Kittelmann et al. (2012) expanded the current database available at NCBI by adding 401 clone sequences obtained from fungal isolates and 183 environmental sequences retrieved from different reports. As a result, the newly curated database contained about 34 clusters and 18 of these clusters contained at least one sequence from a cultured representative thus facilitating taxonomic assignment of unclassified sequences generated from high-throughput sequencing of ARF communities. As the curated database and pipeline is still evolving and not available on the web interface, we were not able to employ the methodology of Kittelmann et al. (2012) for describing taxonomic rank, which we intend to do in further studies. Nonetheless, among the ARF, similar to Liggenstoffer et al. (2010) and Kittelmann et al. (2012), we found *Caecomyces, Cyllamyces, Orpinomyces*, and unclassified genus of *Neocallimastigaceae* to represent the majority of known members of Neocallimastimycota.

It was also evident that the community composition of ARF is determined by diet-induced or host-induced factors (Kittelmann et al., 2012, 2013). Similar to the findings of Boots et al. (2013) and Lima et al. (2015), our results showed that ARF communities were enriched in the rumen of cows fed a higher fiber concentration in the diet, as ARF are known for their significant fibrolytic activity (Ho and Barr, 1995; Orpin and Joblin, 1997). Positive associations between ARF proliferation and dietary forage were also described by Griffith et al. (2010), Kumar et al. (2013), and Mao et al. (2014), similar to this study. The well-characterized genus *Caecomyces* was higher in the D1 (high forage) diet, possibly due to their bulbous rhizoids which can penetrate and expand inside the cellulosic matrix thus accomplishing hydrolysis of plant tissues (Joblin and Naylor, 2010). Other genera such as *Orpinomyces* and *Cyllamyces* were relatively increased with an increased percent of concentrate in the D2 diet, a finding similar to that of Fernando (2012), who reported an abundance of *Orpinomyces* in the bovine rumen fed a high-protein and hay based diet.

### Diversity of Archaea

Methanogenic communities appeared to be resilient to dietary changes while, multiparous cows had slightly more diverse methanogenic populations than primiparous cows. Increasing concentrate percentage in the diet from 25 to 50% did not alter methanogenic communities (Mao et al., 2014) which is similar to our findings. Also, the report of Jeyanathan et al. (2011) concluded that archaeal communities are less diverse and less variable, across species, and diets, than other microbial domains. While there has been several reports describing differences in bacterial community diversity and richness (Jami and Mizrahi, 2012; Li et al., 2012), such comparable data for methanogenic communities are lacking. This might be due to either the low density of archaea (3–4% of total microbes) in the rumen or the establishment of stable methanogenic communities in the rumen at a very early age (Su et al., 2014). Although reports on ruminal methanogens are emerging, the consensus has been that archaea populations contribute only a small proportion (3%) of the rumen microbiome (Belanche et al., 2014). Therefore, further studies are required to determine their abundance as well as their contribution to the host metabolism. In the present study, genus *Methanobrevibacter* was most abundant (>95%), followed by *Methanosphaera* (<5.0%), across all samples. These findings are similar to the earlier reports (Wright et al., 2004, 2006, 2008; Jeyanathan et al., 2011; King et al., 2011; Mao et al., 2014; Seedorf et al., 2014; Singh et al., 2015).

### Co-Occurrence of Bacteria, Anaerobic Rumen Fungi, and Methanogens

The co-occurrence analysis revealed distinct associative patterns across different microbial domains in the rumen of dairy cows. *Prevotella, Paludibacter*, CF231 and *Bacteroidales* from Bacteroidetes, *Lachnospiraceae* from Firmicutes and *Methanobrevibacter* and *Methanosphaera* from archaea showed strong associative patterns both within and between microbial domains indicating their inter-dependence despite perturbations in dietary changes and aging. These taxa may represent the core microbiome communities that could have established in the early stages of life in the rumen and indicate a definitive inter-dependency among bacteria and archaea. It is possible that *Methanobrevibacter* and *Methanosphaera* are dependent on bacterial taxa for their hydrogen needs as they express isoenzymes, methyl coenzyme M reductase (Mcr) I and II, which become active in low and high H_2_ concentrations, respectively (Reeve et al., 1997; Lee et al., 2013). A high dietary fiber concentration can facilitate the proliferation of ARF as well as members of Firmicutes, including the *Ruminococcaceae* and *Succiniclasticum* members, which is revealed not only by the strong interactions within the ARF domain but also between ARF, bacteria and archaea, thus supporting previous reports (Kittelmann et al., 2013). Our study adds new information about the impact of age on co-occurrence patterns among different microbial domains. The ruminal microbial communities in primiparous cows are more homogeneous and tightly clustered and the interactions across different microbial domains is limited to a very few taxa; however, as the cows age, the microbial communities also mature with a myriad of complex microbial interactions as is clearly evident in **Figure 4D**. These intricate relationships among different microbial domains may also contribute significantly to higher feed efficiencies that support higher milk yields in multiparous cows when compared to primiparous cows.

In summary, the present findings showed distinct shifts in the rumen ARF and archaeal communities in both primiparous and multiparous dairy cows fed diets with different forage/concentrate ratios. The co-occurrence analysis revealed a wider representation of the bionetwork among rumen microbial domains that can be influenced by age and diet. However, future studies should focus on better characterization of the unclassified archaeal and anaerobic fungal lineages in the ruminal microbiome using both cultivation-dependent and cultivation-independent approaches. Additionally, metagenomic, and metatranscriptomic studies targeting microbial genomic DNA and RNA are required to gain a better understanding of the microbial ecology and their functional role in dairy cows. Such information can provide greater insights into modulating ration formulations and ultimately can have a positive impact on milk production.

## Conflict of Interest Statement

The authors declare that the research was conducted in the absence of any commercial or financial relationships that could be construed as a potential conflict of interest.
